# African Nightshade (*Solanum scabrum* Mill.): Impact of Cultivation and Plant Processing on Its Health Promoting Potential as Determined in a Human Liver Cell Model

**DOI:** 10.3390/nu10101532

**Published:** 2018-10-17

**Authors:** Grace Akinyi Odongo, Nina Schlotz, Susanne Baldermann, Susanne Neugart, Susanne Huyskens-Keil, Benard Ngwene, Bernhard Trierweiler, Monika Schreiner, Evelyn Lamy

**Affiliations:** 1 Molecular Preventive Medicine, Institute for Infection Prevention and Hospital Epidemiology, University Medical Center and Faculty of Medicine, University of Freiburg, Breisacher Strasse 115b, 79106 Freiburg, Germany; grace.odongo@uniklinik-freiburg.de (G.A.O.); nina.schlotz@uni-konstanz.de (N.S.); 2 Institute of Food Chemistry, Hamburg School of Food Science, University of Hamburg, 20146 Hamburg, Germany; 3 Leibniz Institute of Vegetable and Ornamental Crops, Theodor-Echtermeyer-Weg 1, 14979 Großbeeren, Germany; Baldermann@igzev.de (S.B.); neugart@igzev.de (S.N.); Benard.Ngwene@agcocorp.com (B.N.); Schreiner@igzev.de (M.S.); 4 Department of Food Chemistry, Institute of Nutritional Science, University of Potsdam, Arthur-Scheunert-Allee 114-116, 14558 Nuthetal, Germany; 5 Division Urban Plant Ecophysiology, Faculty of Life Science, Humboldt University Berlin, Lentzeallee 55/57, 14195 Berlin, Germany; susanne.huyskens@hu-berlin.de; 6 Max Rubner-Institut, Federal Research Centre for Nutrition and Food, Institute of Safety and Quality of Fruits and Vegetables, Haid-und-Neu Strasse 9, D-76131 Karlsruhe, Germany; bernhard.trierweiler@mri.bund.de

**Keywords:** aflatoxin B1, African indigenous vegetables, anti-genotoxicity, anti-oxidant activity, cancer chemoprevention, *Solanaceae*

## Abstract

Plant cultivation and processing may impact nutrient and phytochemical content of vegetables. The present study aimed at determining the influence of cultivation and processing on the health promoting capacity of African nightshade (*Solanum scabrum* Mill.) leaves, an indigenous vegetable, rich in nutrients and phytochemicals. Anti-genotoxicity against the human liver carcinogen aflatoxin B1 (AFB_1_) as determined by the comet assay and radical oxygen species (ROS) scavenging capacity of ethanolic and aqueous extracts were investigated in human derived liver (HepG2) cells. ROS scavenging activity was assessed using electron paramagnetic spin resonance and quantification of ARE/Nrf2 mediated gene expression. The cultivation was done under different environmental conditions. The processing included fermentation and cooking; postharvest ultraviolet irradiation (UV-C) treatment was also investigated. Overall, *S. scabrum* extracts showed strong health promoting potential, the highest potential was observed with the fermented extract, which showed a 60% reduction of AFB_1_ induced DNA damage and a 38% reduction in FeSO_4_ induced oxidative stress. The content of total polyphenols, carotenoids and chlorophylls was indeed affected by cultivation and processing. Based on the present *in vitro* findings consumption of *S. scabrum* leaves could be further encouraged, preferentially after cooking or fermentation of the plant.

## 1. Introduction

The broad-leafed African nightshade (*Solanum scabrum* Mill.), locally known as mnavu in Swahili language, is widely used in West, Central and East Africa where it is cultivated as leafy vegetable [1]. The leaves are rich in nutrients, especially proteins, iron, ascorbic acid and riboflavin [2,3] which makes it a very important nutritional source for poor people. During the last years, a rising trend in the use of this crop can be observed as local people become aware of its proclaimed health benefits. A variety of health promoting, bioactive phytochemicals such as phenolic compounds, carotenoids and chlorophylls [2,3] have been identified recently in *S. scabrum*, which could be the reason for its gained popularity in the urban areas but also help explain its long use in traditional medicine. However, it is still unclear how these contribute to human nutrition and health, since scientific evidence on the health promoting activities of *S. cabrum* is scarce.

With increasing demand for this crop in markets, farmers in peri-urban areas have consequently increased its production. The leaves of *Solanum* species are more consumed than the fruits since these can contain high amounts of anti-nutrients such as solanaceous glycoalkaloids like solasodine [4]. For the leaves, this is normally less [4] but they are cooked or fermented with milk to reduce these bitter tasting compounds [4]. Glycoalkaloids have not been detected in *S. scabrum* but in *S. nigrum* and *S. villosum* [4]. Processing conditions including cooking or fermentation, have been reported to impact the phytochemical content of vegetables and fruits [5,6,7,8,9]. Further, this is true for postharvest treatment procedures such as drying or UV-C treatment and also depends on the geographical region [3].

Based on this background, the present study focused on the impact of plant processing on the health promoting, cancer preventive bioactivity of *S. scabrum* leaves. Anti-genotoxicity in terms of DNA protection against aflatoxin B1 and reactive oxygen species (ROS) scavenging capacity was determined using a metabolically competent human liver cell (HepG2) model. The phytochemical content of the differently cultivated and processed *S. scabrum* was also determined to link bioactivity with the phytochemicals in the plant.

## 2. Materials and Methods

### 2.1. Chemicals

Absolute ethanol (EtOH), hydrochloric acid (37%), trypan blue, aflatoxin B1 (purity ≥ 98%), menadione, ethidium bromide and DEAE-Sephadex A-25 were purchased from Sigma-Aldrich Chemie GmbH (Taufkirchen, Germany). Krebs HEPES buffer (KHB), deferoxamine methanesulfonate (DFO), diethyldithiocarbamic acid sodium (DETC) and 1-hydroxy-3-methoxycarbonyl-2,2,5,5-tetramethylpyrrolidine (CMH) were purchased from Noxygen Science Transfer & Diagnostics GmbH (Elzach, Germany). L-glutamine, penicillin and streptomycin were purchased from Invitrogen™ Fisher Scientific GmbH (Schwerte, Germany). Dulbecco’s modified Eagle’s medium (DMEM), foetal calf serum (FCS), trypsin 10* (25 mg/mL), trypsin-EDTA 10* (5 mg/mL and 2.2 mg/mL) and phosphate buffered saline (PBS, without Ca and Mg) were purchased from Gibco™, Life Technologies GmbH (Darmstadt, Germany). Triton-X 100 was from Carl Roth GmbH & Co. KG (Karlsruhe, Germany) and dimethyl sulfoxide (DMSO; purity > 99%) was purchased from Applichem GmbH (Darmstadt, Germany). Low melting point agarose (LMPA) and normal melting point agarose (NMPA) were purchased from Serva GmbH (Heidelberg, Germany).

The following chemicals and reagents were used for chemical analyses: Methanol (99.95%), ammonium acetate (Carl Roth GmbH & Co. KG, Karlsruhe, Germany); tetrahydrofuran (99.7%; VWR International GmbH, Darmstadt, Germany); methyl tert-butyl ether (99.8%; Chemsolute, Th. Geyer GmbH & Co. KG, Renningen, Germany); dichloromethane (99.9%), isopropanol (99.95%) and zeaxanthin were purchased from Carote Nature GmbH (Ostermundigen, Switzerland); β-carotene, lutein and chlorophylls a & b were from Sigma-Aldrich Chemie GmbH (Taufkirchen, Germany). Chlorogenic acid, quercetin 3-*O*-glucoside, kaempferol 3-*O*-glucoside and isorhamnetin-3-*O*-glucoside were from Carl Roth GmbH and Co. KG (Karlsruhe, Germany). Ferrous sulphate heptahydrate (FeSO_4_·7H_2_O) was purchased from Merck Chemicals GmbH (Darmstadt, Germany). Arylsulfatase, isolated from *Helix pomatia*, was purchased from Roche Diagnostics GmbH (Mannheim, Germany).

### 2.2. Solanum scabrum

Seeds of *S. scabrum* were provided by the World Vegetable Centre (AVRDC) and cultivated at four different locations; the Max Rubner Institute, Federal Research Institute of Nutrition and Food, Karlsruhe, Germany (location A; 49°0′50.976″ N 8°25′35.832″ E and altitude 116 m above sea level), the Leibniz Institute of Vegetable and Ornamental Crops Großbeeren (IGZ) Großbeeren, Germany (location B; 52° latitude North, 13° longitude East and altitude 43 m above sea level), Humboldt University, Berlin, Germany (location C; 52° latitude North, 13° longitude East and altitude 43 m above sea level) and at Jomo Kenyatta University of Agriculture and Technology (JKUAT), Nairobi, Kenya (location D; latitude 1°18′ N; longitude 37°12′ N).

Growth conditions at the different location was as follows; at location A, *S. scabrum* was cultivated using peat-based substrate (Gramoflor) with the following specifications: pH (CaCl_2_) 5.8; N (CaCl_2_—mg/L) 140; P_2_O_5_ (Cal—mg/L) 160; K_2_O (Cal—mg/L) 180. The plants were grown in a climatic chamber for four weeks at day/night temperatures of 25/20 °C, relative humidity of 40/70% and light for 12.5 h. After four weeks, plants were transferred to the greenhouse and kept there under ambient temperature (according to seasonal influences up to 40 °C in the summer) and without humidity control for three weeks.

At location B, seeds of *S. scabrum* were sown in 5 L plastic pots containing standard potting substrate (Einheitserdewerke Werkverband e.V., Sinntal-Altengronau, Germany) with the following specifications: pH (CaCl_2_) 5.8; KCl (g/L) 2; N (CaCl_2_—mg/L) 340; P_2_O_5_ (Cal—mg/L) 380; K_2_O (Cal—mg/L) 420. The plants were grown in a greenhouse for seven weeks after germination with sufficient irrigation. During this period, average day/night temperature was 21/18.6 °C respectively with an average air humidity of 54%. Additional cultivation experiment was done by growing some plants in an open green house. During harvest, fully developed leaves were collected in triplicate, each replicate consisting of 200 g of fresh leave material pooled from about ten plants. The leaves were then either immediately freeze-dried (control), or processed for further treatments (fermentation or thermal processing or UV treatment). At location C, the seeds of *S. scabrum* were grown under greenhouse conditions at the experimental station in Berlin-Dahlem, with the temperature, relative air humidity and light intensity during the experiments continuously recorded. Eight weeks after sowing, leaves were harvested and immediately subjected to UV-C treatments.

At Location D, seeds of *S. scabrum* were sown directly on an experimental field plot with a spacing of 20 × 30 cm. The soil is described as a clay soil with a pH of about 5.2. Mean annual rainfall in Juja, Nairobi, is about 1500 mm and temperature of about 20 °C averaged the years 2000–2012 according to World weather online (2016). Before planting, the plot was supplied with 2 kg/m^2^ well-decomposed manure and 5 g di-ammonium phosphate per planting hole. Two weeks after planting, the seedlings were additionally fertilized with 6 g calcium ammonium nitrate per plant as recommended based on soil analyses. During the course of the experiment, the plants were irrigated manually with tap water twice a day. Plants were grown for 6 weeks. During harvest (8–12 leaf stage), fully developed leaves were collected in duplicate, each replicate consisted of about 300 g of fresh leaf material pooled from about 10 plants selected randomly on the field. The leaves were then freeze-dried and transported to IGZ Großbeeren, Germany, for chemical analyses.

**Figure 1:**
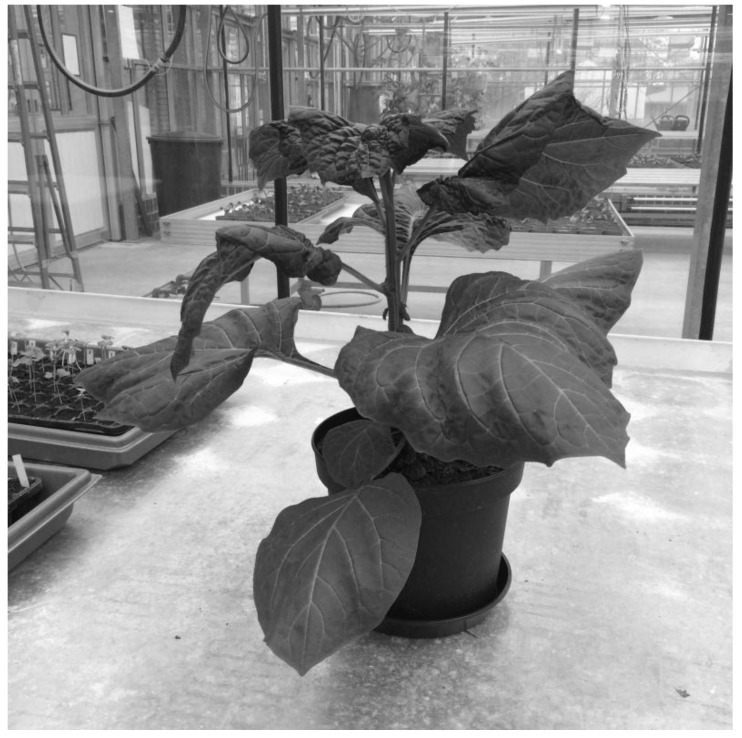
*S. scabrum* aerial parts.

The aerial parts of *S. scabrum* Mill are shown in Figure 1 below.

#### Processing of *S. scabrum* Leaf Material

Fermentation: this was done by submerged fermentation where freshly harvested leaves; 700 g (location A) were washed with tap water, then immersed in 10 L crock pots typically used in Germany for Sauerkraut fermentation as previously described [10,11], with 2.1 L of a 2.5% brine solution (containing 3% salt and 3% sugar). The fermentation was inoculated with 1 × 10^7^ CFU/mL of each of the starter bacteria *Lactobacillus plantarum* BFE 5092 and *Lactobacillus fermentum* BFE 6620 and left to ferment at 25 °C for 144 h. This fermentation was followed by removal of the brine, weighing of the plant material, freeze drying and grinding to a fine powder.

Cooking (thermal treatments): this entailed boiling of the leaf material where freshly harvested leaves (location B) were washed with tap water then sliced to 1 cm pieces with a kitchen knife and then finally immersed in 100 mL of boiling water. They were cooked (simmered) for 20 min and then the excess water was drained out using a sieve. The drained samples were immediately cooled on ice and then frozen. They were freeze dried and ground to a fine powder.

UV-C treatment: The freshly harvested leaves of *S. scabrum* were immediately treated with UV-C in an UV-C chamber (ABOX^®^ UV Technology, UMEX GmbH, Germany), where temperature and relative air humidity were kept constant at 5 °C and 85%, respectively. UV-C dosage was achieved with medium pressure mercury vapour discharge lamps with a peak emission at 254 nm (VL-6C, 6 W–254 nm Tube, Power: 11 W, Vilber Lourmat GmbH, Germany). The lamps were placed at a distance of 0.4 m to leaves. The dosage was calculated from the product of exposure time and irradiance, as measured by a portable handheld digital radiometer (UVPAD-E, Opsytec Dr. Gröbel GmbH, Germany). Based on this, two different dosages were applied, that is, 1.7 kJ m^−2^ and 3.4 kJ m^−2^. Non-treated plants served as control. Thereafter, leaves were stored at 5 °C (85% relative Humidity: RH) for 14 days and 20 °C (85% RH) for 6 days. The leaves were then freeze dried and ground to fine powder.

### 2.3. Extract Preparation by Sonication

The solvents used for extraction of the freeze-dried plant powder were either 70% ethanol (EtOH) or double distilled water (ddH_2_O) each used at a 1:10 ratio. A sample of 0.5 g of the plant powder was diluted in 5 mL of 70% EtOH or ddH2O (stock concentration: 100 mg/mL). The mixture was incubated using a sonicator water bath at 50 °C for 30 min as previously described [12] and was sterile filtered using a 0.22 µm MillexR syringe-driven filter unit (fast flow and low binding Millipore). Then, 1:3 serial dilutions were made with 70% EtOH or ddH_2_O from the stock solution. The cells were subsequently treated with the dilutions at a 1:100 ratio resulting in final extract concentrations starting from 333 to 1.4 µg/mL. In each of the experiments, fresh extracts were always prepared from the freeze-dried leaf powder.

### 2.4. Determination of S. scabrum Phytochemical Content

Chemical analyses were done as previously described [12,13]. Phenolic compounds were analysed by high pressure liquid chromatography (HPLC) (Agilent HPLC series 1100, Agilent Technologies Sales & Services GmbH & Co. KG, Waldbronn, Germany) coupled to an ion trap mass spectrometer (Bruker Amazon SL, Bruker, Bremen, Germany). Quantification of phenolics was done using standards of caffeoylquinic acid [chlorogenic acid], quercetin 3-*O*-glucoside, kaempferol 3-*O* glucoside and isorhamnetin-3-*O*-glucoside (Carl Roth, Karlsruhe, Germany), which were used for external calibration curves. Carotenoids and chlorophylls were analysed using ultra-high-pressure liquid chromatography (UHPLC) (Agilent Technologies 1290 Infinity II) coupled with time of flight mass spectrometry (ToF-MS) (Agilent Technologies 6230 TOF LC/MS) equipped with an atmospheric pressure chemical ionization source (Agilent Technologies). External standard calibration curves of each compound were used for quantification. Compounds below the quantification limit were indicated as not detected (n.d.).

### 2.5. Cell Cultures

The cell lines used were human derived liver (HepG2) cells and a genetically modified form (ARE reporter HepG2 cells). The HepG2 cell line (ACC-180) was obtained from the German Collection of Microorganisms and Cell Cultures (DSMZ; Braunschweig, Germany) while the recombinant ARE reporter–HepG2 cell line designed to monitor the nrf2 antioxidant response pathway was obtained from BPS Bioscience, Inc., (San Diego, Califonia, USA) (60513-GVO-BPS). The cells were cultured as described [12] with few modifications; the cells were grown in DMEM medium supplemented with 15% FCS and 1% penicillin/streptomycin solution. Additionally, 600 µg/mL geneticin antibiotic was used in ARE reporter HepG2 cell line. Incubation was done in a 95% humidified incubator at 37 °C and 5% CO_2_.

### 2.6. Assessment of Anti-Genotoxic Activity of Leaves of S. scabrum Using the Comet Assay

The Comet assay was used to determine anti-genotoxic activity of *S. scabrum* which was performed according to Lamy et al. [14], with few modifications. Pre-treatment of HepG2 cells was done with either water or ethanol extracts from *S. scabrum* for 24 h, experimental control were considered. The cells were then washed and exposed to 10 µM AFB_1_ or 0.1% DMSO for another 24 h. Comet assay was then performed and analysis done with Comet 5.5 image software (Munich, Germany) connected to a Leica fluorescence microscope (Leica DMLS; Wetzlar, Germany; excitation filter: BP 546/10 nm; barrier filter: 590 nm) connected to a high sensitivity charge-coupled (CCD) camera. The percent tail DNA was used as the parameter for indicating DNA damage.

### 2.7. Determination of Anti-Oxidant Activity of S. scabrum

The anti-oxidant activity of *S. scabrum* was determined by electron paramagnetic resonance spectroscopy (EPR) equipped with temperature and gas controller Bio III (Noxygen, Elzach, Germany) which is capable of detecting ROS in the cells. HepG2 cells pre-treated with water or ethanolic extracts from *S. scabrum* were used. The EPR spectroscopy protocol previously described by Lamy et al. [15] was adopted with few modifications. HepG2 cells were exposed to (a) 200 µM menadione or 0.1% DMSO (solvent control) for 30 min or (b) 100 µM ferrous sulphate or supplemented DMEM medium (solvent control) for 15 min. The cells were then washed with pre-warmed Krebs-HEPES buffer (KHB) followed by either 30 min incubation at 37 °C with spin probe 100 µM CMH in KHB supplemented with 25 µM DFO and 5 µM DETC, or 120 min incubation at 37 °C with 1-hydroxy-4-phosphono-oxy-2,2,6,6-tetramethyl-piperidinethe (PPH) at a concentration of 200 µM in KHB supplemented as mentioned above. Supernatants were transferred to new reaction tubes and kept on ice. A 10 scan screening was done for each sample using 50 µL glass capillaries.

### 2.8. Assessing Induction of Nrf2 Antioxidant Pathway by S. scabrum

The ARE/Nrf2 reporter gene activity was measured to determine the induction of the Nrf2 anti-oxidant pathway which was analysed according to the manufacturer’s instructions using the ONE-Glo™ Luciferase Assay System (Promega GmbH, Mannheim, Germany). In brief, HepG2-ARE cells were seeded in 96 well plates (4 × 10^4^ cells/well) and immediately exposed to the *S. scabrum* extracts. After incubation for 18 h, cells were lysed and luminescence measured 15 min after substrate addition using an infinite M200 microplate reader (Infinite M200, Tecan Group Ltd., Männedorf, Switzerland).

### 2.9. Measurement of Cytotoxicity and Cytostatic Activity

Cytotoxicity was determined by checking the viability of HepG2 cells treated with *S. scabrum* extract or solvent control after 48 h using the trypan blue dye exclusion test. Total numbers of extract treated cells were compared with the solvent control. Counting was done using an Neubauer improved counting chamber (Brand GmbH & Co. KG, Wertheim, Germany).

### 2.10. Data Analysis

All data were analysed using Graph Pad Prism 6 (GraphPad Software Inc., San Diego, CA, USA). Results of the cell culture experiments are presented as means of at least three independent experiments. Differences were considered significant at *p* ≤ 0.05 (*), *p* ≤ 0.01 (**). Statistical significance was assessed using two-way analysis of variance (ANOVA) followed by Dunnett’s multiple comparisons test.

## 3. Results

### 3.1. Phytochemical Composition of Raw, Processed and UV-C Treated S. scabrum

The impact of cultivation and processing condition on the content of secondary plant metabolites is presented in Table 1, Table 2, Table 3, Table 4 and Table 5. Phenolic compounds, carotenoids and chlorophylls were analysed in the plant extracts. The highest amounts of phenolic compounds (19,644.4 µg/mL) or (196,444 µg/g dry matter (DM) of leaves) were detected in raw extracts from plants grown in open greenhouse from location B (see Table 4). The least was found in the cooked ethanolic extracts from location B (3024.2 µg/mL) or (30,242 µg/g DM) (see Table 2). Carotenoids and chlorophylls were not detected or only at minor amounts in water extract (see Table 1 and 2) but in ethanolic extracts, the content of carotenoids ranged from 17 µg/mL or 170 µg/g DM (raw plant extracts from open greenhouses) to 179 µg/mL or 1790 µg/g DM (fermented plant extracts). The chlorophyll content ranged from 5 µg/mL or 50 µg/g DM in the fermented plant extract to 464 µg/mL or 4640 µg/g DM in the raw plant extract from location C.

### 3.2. Effect of Cultivation and Processing on the Protective Potential of S. scabrum against AFB_1_ Induced DNA Damage

**Figure 2:**
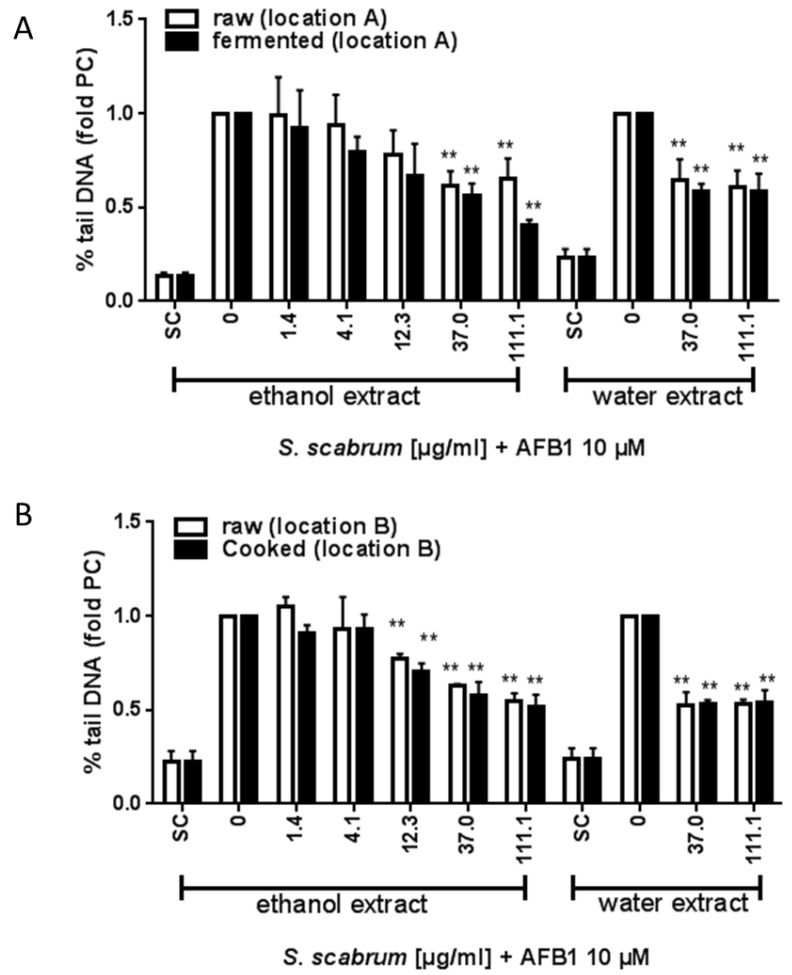
Anti-genotoxic activity of raw and processed *S. scabrum*. Results derived from the Comet assay are shown as percent tail DNA (**A**,**B**) calculated relative to AFB_1_-treated cells. SC: solvent control, 0.1% DMSO + 0.7% ethanol (for ethanolic extracts) or 0.1% DMSO + ddH_2_O (for water extracts). Data are means ± SEM of three independent experiments. Asterisks indicate statistically significant differences between the respective treatment and the positive control (without *S. scabrum* leaf extract) *p* ≤ 0.01 (**).

**Figure 3:**
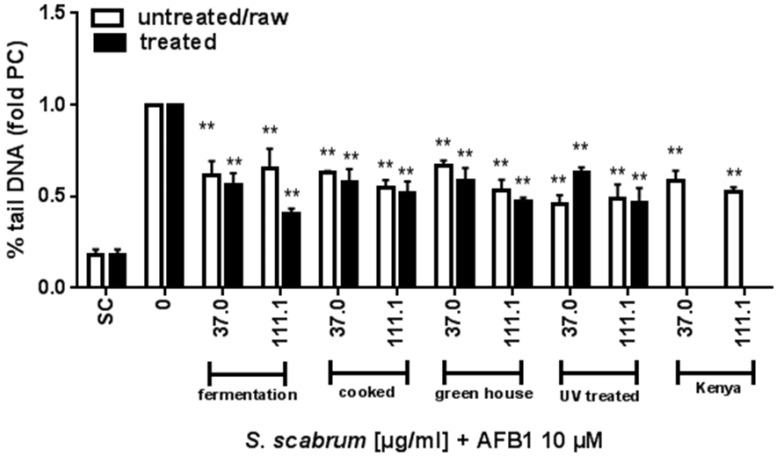
Impact of cultivation, processing and UV-C on the anti-genotoxic activity of *S. scabrum* against AFB_1_. Results derived from the Comet assay are shown as percent tail DNA calculated relative to AFB_1_-treated cells. SC: solvent control, 0.1% DMSO + 0.7% ethanol. Data are means ± SEM of three independent experiments. Asterisks indicate statistically significant differences between the respective treatment and the positive control (without *S. scabrum* leaf extract) *p* ≤ 0.01 (**).

The effect of *S. scabrum* extracts against aflatoxin induced DNA damage was assessed using the comet assay. Basically, all the extracts tested (different cultivation environment and processing conditions) were protective against AFB_1_ induced damage by more than 30% at the highest concentration tested (111 µg/mL) as shown in Figure 2 and Figure 3. The anti-genotoxic potential against AFB_1_ of raw extracts from plants cultivated within locations in Germany varied between 34 to 51% (Germany, location A: 34%, location B; 45%, location C: 51%) or Kenya (48%). Cultivation under open or normal greenhouse conditions did not significantly affect the anti-genotoxic potential (open greenhouse: 53% vs. normal greenhouse: 47%), Figure 3. Overall, processing in terms of fermentation or cooking further increased the anti-genotoxic potential of the plant extract as compared to the raw material (fermented: 60% vs. raw: 34%; cooked: 49% vs. raw: 45%). With 60% inhibition, the fermented ethanolic extract demonstrated the strongest protection against AFB_1_ (see Figure 2A).

When plants were treated with UV-C after harvesting, the anti-genotoxic potential was less at 37 µg/mL as compared to the untreated plant material but this was compensated for at the highest concentration tested (UV-C treated: 53% vs. control 51%), Figure 3. The results derived by water extraction were comparable to ethanol extraction (Figure 2).

### 3.3. ROS Scavenging Activity and Induction of ARE/Nrf2-Mediated Gene Expression

**Figure 4:**
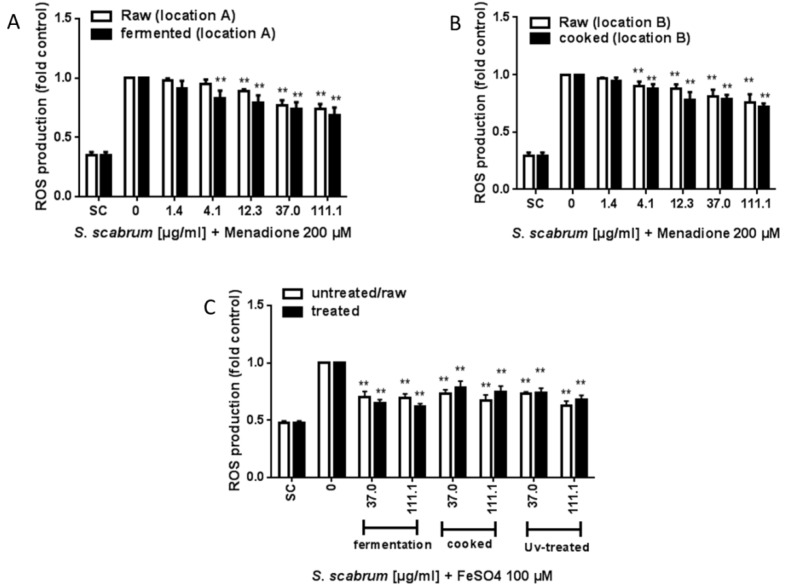
Anti-oxidant activity of raw, processed and UV-C treated ethanolic *S. scabrum* extracts. Inhibition of ROS production was determined by the EPR method in response to 200 µM menadione (**A**,**B**) or 100 µM FeSO_4_ (**C**) in HepG2 cells. Data are means ± SEM of three independent experiments expressed as fold control (SC: solvent control, 0.1% DMSO + 0.7% ethanol (**A**,**B**) or supplemented DMEM medium (**C**)) + 0.7% ethanol). Asterisks indicate statistically significant differences between the respective treatment and the positive control (without *S. scabrum* leaf extract) *p* ≤ 0.01 (**).

First, the capacity of ROS scavenging by the plant extracts was investigated using EPR. ROS production by menadione or FeSO_4_ resulted in a 4-fold or 2.2-fold increase, respectively over the solvent control (data not shown). In both experimental settings either using menadione or FeSO_4_ as stressor, all the tested extracts showed the potential to reduce stimulated ROS production (Figure 4 A–C). At the highest concentration tested, reduction in ROS production triggered by menadione ranged between 26% by plant extracts from location A (Figure 4A) to 24% from location B (Figure 4B). Processing either by fermentation or cooking did not impact the antioxidant activity as compared to the raw extract (fermented extract: 31% reduction, cooked extract: 28% reduction). A similar observation was made for ROS inhibition triggered by FeSO_4_ (Figure 4C) although the inhibitory potency was somewhat higher compared to that seen with ROS inhibition induced by menadione. Inhibition was 38% by the fermented extract from location A, 26% by the cooked extract from location B and 38% by the UV-C treated extract from location C (Figure 4C).

**Figure 5:**
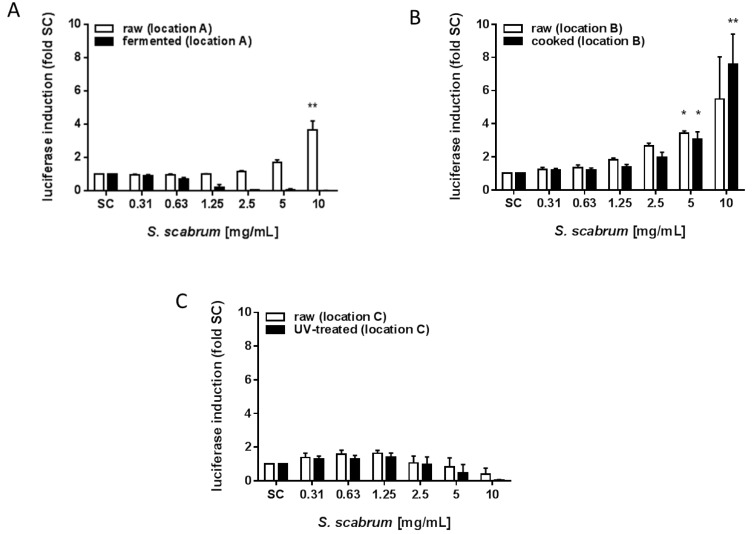
Induction of ARE/Nrf2- mediated gene expression by ethanolic extracts of *S. scabrum* (raw, processed and UV-C treated). Results are fold induction of luciferase as indicator of ARE/Nrf-2-mediated gene expression. Luciferase induction is given for (**A**) raw vs. fermented, (**B**) raw vs. cooked and (**C**) raw vs. UV-treated *S. scabrum* leaves. Data are means ± SEM of three independent experiments expressed as fold control (SC: solvent control, 0.7% ethanol). Asterisks indicate statistically significant differences between the respective treatment and the solvent control *p* ≤ 0.05 (*) *p* ≤ 0.01 (**).

Additionally, the potential of the extracts to activate the Nrf2 antioxidant pathway was investigated using a Nrf2/ARE Luciferase Reporter HepG2 cell line (Figure 5A–C). In general, the effect of the extracts in this assay was very weak. Only after 100 times concentration of the plant extract, gene expression could be seen in this assay. Then, a concentration-dependent increase in ARE/Nrf2- mediated gene expression was observed upon exposure to the raw extract from location A, with an up to 4-fold induction (Figure 5A) but not after processing in terms of fermentation. Further, extracts prepared from raw and cooked plant material from location B (Figure 5B) showed a 6-fold and 8-fold increase at the highest concentration tested, respectively. In contrast to this, plant material from location C, either untreated or processed had no effect in the assay (Figure 5A,C).

### 3.4. Induction of Cytotoxicity by Ethanolic S. scabrum Extracts

**Figure 6:**
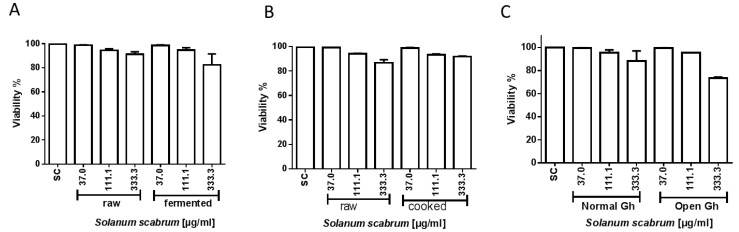
Cytotoxicity of ethanolic extracts of *S. scabrum*. HepG2 cells were treated for 48 h with the leaf extracts and cytotoxicity determined using trypan blue dye exclusion test. Results are given for (**A**) raw vs. fermented, (**B**) raw vs. cooked and (**C**) normal greenhouse vs. open green house experiments. Data are means ± SEM of three independent experiments (SC = solvent control, 0.7% ethanol).

Relevant cytotoxicity could not be observed upon treatment of the cells (HepG2) with the extracts of *S. scabrum* for 48 h as shown in Figure 6A-C. All the concentrations of the extract tested showed a viability >70%.

## 4. Discussion

The cancer preventive potential in terms of protection from DNA damage and oxidative stress of *Solanum* species has previously been studied but not much information has been available for the leaves. Anti-mutagenicity as determined by the micronucleus test in mice bone marrow was shown by ethanolic fruit extracts from *S. lycocarpum* [16] and ethanolic leaf extracts from *S. paniculatum* L. [17]. Aqueous leaf extract of *S. nigrum* have been shown to suppress mitochondrial function and epithelial mesenchymal transition in MCF-7 breast cancer cells [18] and to cause >50% cytotoxicity in human A-375 melanoma cells [19]. The present in vitro findings suggest that basically, at non-toxic concentrations, the leaf extract of *S. scabrum* has chemopreventive properties in terms of anti-genotoxicity against AFB_1_ and antioxidant potential. The cultivation conditions of the plant obviously had an impact on the observed anti-genotoxic potential against AFB_1_ but with an observed variance of 17% it was not that marked between the raw extracts derived from different locations. This information is important because it shows that no matter if the plants were grown under open field conditions in the climate region of Germany or Kenya or under very controlled conditions such as greenhouse usage, a basic health promoting potential of the plant leaves seems to be given. Currently, there exist no standard procedure for cultivation and processing of African indigenous vegetables (AIVs). However, it is an area that is slowly drawing interest with the focus of trying to come up with the optimal cultivation conditions and processing method. So far, pre- and post-harvest procedures have been reported to impact the content of phytochemicals of the AIVs and could consequently also affect the bioactivity. As an example, the leaf extract from the medicinal plant *Achillea collina* grown in high altitude had a higher polyphenol level than the one grown in low altitude [20]. This was also shown for the soil water content [21]. For plant fermentation, it has been shown to impact the polyphenolic content and then also the antioxidant activity [22,23]. Thus, we addressed this issue for *S. scabrum* in the present study.

Now, when comparing the results on anti-genotoxicity with the data from chemical analysis, a few facts are remarkable. First, the least potent raw extract (location A) showed a reduction in AFB_1_-induced genotoxicity by 34%, the most potent one (location C) by 51%. However, the total polyphenol content of the latter one was about 30% less as compared to the one from location A, carotenoids and chlorophylls were present in both extracts at the same range. Second, there was not much difference between the bioactivity of the raw and processed extracts in terms of cooking or fermentation. The anti-genotoxic potential of raw and fermented plant extracts was comparable or rather increased by fermentation. However, they differed in their total polyphenol content by about 70%, whereas the fermented extract contained the minor amount of both. The two extracts did also not contain any or only low amounts of carotenoids and chlorophylls. Third, raw and cooked plant extracts were equally potent in the anti-genotoxicity assay but then, the raw plant extract contained only 50% of the total polyphenols compared to the cooked plant extract. Fourth, water and ethanolic extracts showed a comparable anti-genotoxicity even though their phytochemical content differed strongly in all analysed groups.

Polyphenols are well known for their anti-genotoxic, antioxidant and anti-cancer capacities [24,25,26]. These phytochemicals also can activate the NRF2 pathway and in the end result in production of antioxidant enzymes capable of reducing oxidative stress in the cells [27,28,29]. The analysed carotenoids contained majorly lutein and zeaxanthin which have previously been shown to have antioxidant activity [29,30,31,32] and also chlorophylls are known anti-genotoxicants and antioxidants [33]. Carotenoids have also been reported to interact synergistically with polyphenols to confer antioxidant activity [34]. In the present study, all tested extracts showed antioxidant capacity against both menadione and FeSO_4_ with a comparable potential. While menadione induces ROS by redox cycling via one-electron reductive enzymes [35], the iron (II) ion from FeSO_4_ acts as key generator in the intracellular formation of ROS such as H_2_O_2_ and OH radicals [36,37]. In another study by our group we could detect significant antioxidant activity of the AIV *Amaranthus cruentus* against FeSO_4_ but not menadione [38]. Due to the differential finding, we then argued that indeed the detected polyphenols in the plants could account for the observed anti-oxidant effect since their antioxidant activity largely depends on their iron-chelating properties [39]. This similarly could explain the activity seen here with *S. scabrum* against FeSO_4_.

Taken together, for the present study, no clear phytochemical pattern was identified, which could sufficiently explain the observations in bioactivity. For the AIV *B. carinata*, we already found that plant processing in terms of cooking or fermentation had an impact on the phytochemical content but this did not adversely affect the anti-genotoxicity of the plant, too [12]. Cooking then rather increased the antioxidant capacity of the ethanolic plant extract. Of course, synergistic effects of the diverse bioactive compounds as well as activity of de-glucosylated and decomposition products could also have some impact. As previously studied [40,41], the radical scavenging activity of polyphenols was reported to be stable although the original compound mixture was decomposed. In another study, synergy between compounds was proposed to be up to 24% of the observed antioxidant activity which could be explained by the calculated total phenolic compounds from red wine [42]. Besides polyphenols, carotenoids and chlorophylls [3,43], *Solanum* species were reported to possess a number of further potent bioactive phytochemicals such as solasodine, solanidine and solamargine that also have anticancer and antioxidant activity [44,45,46] but these have not been analysed here.

Photosynthetically ineffective light such as UV light has increasingly gained interest for improving postharvest quality [9], at present primarily UV-C (190–280 nm) applied for effective surface decontamination [47,48], directly damaging microbial DNA. However, it also affects textural properties [49] and induces the biosynthesis of plant secondary metabolites [9,50,51]. Thus, the present investigations also analysed whether this postharvest method might be recommended also in terms of bioactivity preservation. Present results confirmed that neither the anti-genotoxic nor antioxidant potential of the plant is adversely affected. Thus, UV-C treatment might be recommended when envisaging postharvest disinfection purposes, that is, food safety issues of AIVs in postharvest and guaranteeing no negative impact on health promoting compounds.

The present study was carried out using a human liver-derived cell line which is known for their metabolically competence in phase I and II enzyme activities [52,53]. Thus, it is a useful in vitro system to partly reflect the human liver metabolism of the phytochemicals and also the applied carcinogen AFB_1_. However, of course it cannot reflect the complex processes that occur during digestion of the plant material in the human body. Especially the gut microbiota is known to extensively metabolize phytochemicals which in consequence greatly influences their bioavailability [54]. This is certainly a limitation of the study.

## 5. Conclusions

Irrespective of the processing method, consumption of the leaves from *S. scabrum* might help in cancer prevention strategies, based on the present in vitro findings. Processing clearly affected the content of phytochemicals but this did not result in a diminished chemopreventive effect. However, other endpoints relevant for cancer prevention may perhaps be influenced by this change in phytochemicals, in a negative or positive way. Cooking and fermentation are simple preservation methods in terms of preventing the growth of bacteria, fungi and other microorganisms, which gives them a clear advantage to consumption of the raw plant material. Selection of the *Solanum* species for consumption should be carefully considered since species like *S. nigrum* have been reported to have toxic substances in certain part of the plants. The cheap UV-C postharvest treatment could be a potential alternative based on the present findings.

## Figures and Tables

**Table 1:** Phytochemical content in the extracts of raw vs. fermented *S. scabrum* in µg/g DM (location A). The data are presented as phytochemical content ± average analytical error.

Polyphenols	Raw Ethanol	Raw Water	Fermented Ethanol	Fermented Water
tentative structure				
3-caffeoylquinic acid	1314 ± 26	828 ± 41	1186 ± 24	1493 ± 30
5-caffeoylquinic acid	1023 ± 20	1365 ± 27	1490 ± 30	2315 ± 46
4-caffeoylquinic acid	30,839 ± 617	8311 ± 166	5394 ± 108	15,032 ± 301
caffeoylmalate	n.d.	1574 ± 31	3688 ± 74	1418 ± 28
caffeoylmalate	52,628 ± 1053	34,750 ± 695	12,527 ± 251	35,211 ± 704
quercetin-3-glucosylrhamnogalcatoside	185,237 ± 3704	2083 ± 42	479 ± 24	994 ± 50
coumaric acid	1622 ± 32	653 ± 33	850 ± 43	533 ± 27
quercetin-3-rhamnogalactoside	422 ± 21	438 ± 22	n.d.	n.d.
quercetin-3-rhamnosylrhamnogalactoside (isomer 2)	3326 ± 67	5475 ± 110	1776 ± 36	5428 ± 109
quercetin-3-rhamnosylrhamnogalactoside (isomer 3)	1649 ± 33	2396 ± 48	932 ± 47	2930 ± 59
sinapoylmalate	608 ± 30	644 ± 32	566 ± 28	809 ± 40
kaempferol-3-diglucoside	423 ± 21	643 ± 32	538 ± 27	1036 ± 21
kaempferol-3-rhamnosylrhamnogalactoside (isomer 1)	528 ± 26	868 ± 43	564 ± 28	589 ± 29
quercetin-3-pentosylrutinoside	1787 ±36	2090 ± 42	1024 ± 20	2661 ± 53
sinapic acid	1705± 34	539 ± 27	1131 ± 23	2742 ± 55
kaempferol-3-rhamnosylrhamnogalactoside (isomer 1)	892 ± 45	688 ± 34	610 ± 31	1004 ± 20
quercetin-3-rutinoside	1346 ± 27	709 ± 35	911 ± 46	1737 ± 35
Total	101,964 ± 2039	64,054 ± 1281	33,666 ± 673	75,343 ± 1507
**Carotenoids**				
β-carotene	1 ± 0.1	n.d.	1 ± 0.1	n.d.
zeaxanthin	70 ± 7.0	1 ± 0.1	1326 ± 132.6	4 ± 0.4
lutein	515 ± 51.5	1 ± 0.1	462 ± 46.2	n.d.
Total	586 ± 58.6	2 ± 0.2	1789 ± 178.9	4 ± 0.4
**Chlorophylls**				
chlorophyll a	3500 ± 350.0	n.d.	n.d.	n.d.
chlorophyll b	1076 ± 107.6	n.d.	50 ± 5.0	n.d.
Total	4576 ± 457.6	n.d.	50 ± 5.0	n.d.

(n.d. = not detected). Phytochemical classes are put in bold.

**Table 2:** Phytochemical content in the extracts of raw vs. cooked *S. scabrum* in µg/g DM (location B). The data are presented as phytochemical content ± average analytical error.

Polyphenols	Raw Ethanol	Raw Water	Cooked Ethanol	Cooked Water
tentative structure				
3-caffeoylquinic acid	1390 ± 28	1626 ± 33	1314 ± 26	1879 ± 38
5-caffeoylquinic acid	1231 ± 25	1637 ± 33	1409 ± 28	2273 ± 45
4-caffeoylquinic acid	4946 ± 99	4630 ± 93	2460 ± 49	4567 ± 91
caffeoylmalate	800 ± 40	3226 ± 65	702 ± 35	2513 ± 52
caffeoylmalate	31,178 ± 624	65,157 ± 1303	22,048 ± 441	58,559 ± 1171
quercetin-3-glucosylrhamnogalcatoside	611 ± 31	734 ± 37	548 ± 27	611 ± 31
coumaric acid	598 ± 30	913 ± 46	650 ± 33	811 ± 41
quercetin-3-rhamnogalactoside	367 ± 18	247 ± 12	n.d.	n.d.
quercetin-3-rhamnosylrhamnogalactoside (isomer 2)	901 ± 18	1198 ± 24	n.d.	856 ± 43
quercetin-3-rhamnosylrhamnogalactoside (isomer 3)	541 ± 27	796 ± 40	n.d.	629 ± 31
sinapoylmalate	520 ± 26	540 ± 27	n.d.	n.d.
kaempferol-3-diglucoside	n.d.	423 ± 21	n.d.	n.d.
kaempferol-3-rhamnosylrhamnogalactoside (isomer 1)	430 ± 22	506 ± 25	398 ± 20	445 ± 22
quercetin-3-pentosylrutinoside	319 ± 16	369 ± 18	272 ± 14	309 ± 15
sinapic acid	n.d.	n.d.	n.d.	n.d.
kaempferol-3-rhamnosylrhamnogalactoside (isomer 1)	376 ± 8	387 ± 8	n.d.	n.d.
quercetin-3-rutinoside	518 ± 26	497 ± 25	441 ± 22	393 ± 20
Total	44,726 ± 895	82,886 ± 1658	30,242 ± 605	73,845 ± 1477
**Carotenoids**				
β-carotene	1 ± 0.1	n.d.	1 ± 0.1	1 ± 0.1
zeaxanthin	50 ± 5.0	n.d.	41 ± 4.1	1 ± 0.1
lutein	640 ± 64.0	n.d.	355±35.5	6 ± 0.6
Total	691 ± 69.1	n.d.	397 ± 39.7	8 ± 0.8
**Chlorophylls**				
chlorophyll a	1991 ± 199.1	n.d.	898 ± 89.8	17 ± 1.7
chlorophyll b	1272 ± 127.2	n.d.	583 ± 58.3	n.d.
Total	3263 ± 326.3	n.d.	1481 ± 148.1	17 ± 1.7

(n.d. = not detected). Phytochemical classes are put in bold.

**Table 3:** Phytochemical content of extracts of untreated vs. UV-C treated *S. scabrum* in µg/g DM (location C). The data are presented as phytochemical content ± average analytical error.

Polyphenols	Untreated Ethanol	UV-C Treated Ethanol
tentative structure		
3-caffeoylquinic acid	1658 ± 33	1042 ± 21
5-caffeoylquinic acid	4009 ± 80	4715 ± 94
4-caffeoylquinic acid	34,633 ± 693	38,998 ± 780
caffeoylmalate	820 ± 16	775 ± 39
caffeoylmalate	18,594 ± 372	26,400 ± 528
quercetin-3-glucosylrhamnogalcatoside	1928 ± 39	2490 ± 50
coumaric acid	1215 ± 24	784 ± 39
quercetin-3-rhamnogalactoside	388 ± 19	439 ± 22
quercetin-3-rhamnosylrhamnogalactoside (isomer 2)	2912 ± 58	3436 ± 69
quercetin-3-rhamnosylrhamnogalactoside (isomer 3)	1546 ± 31	1832 ± 37
sinapoylmalate	571 ± 29	612 ± 31
kaempferol-3-diglucoside	433 ± 22	475 ± 24
kaempferol-3-rhamnosylrhamnogalactoside (isomer 1)	574 ± 29	594 ± 30
quercetin-3-pentosylrutinoside	1126 ± 23	1332 ± 27
sinapic acid	1431 ± 29	1329 ± 27
kaempferol-3-rhamnosylrhamnogalactoside (isomer 1)	651 ± 33	628 ± 31
quercetin-3-rutinoside	1045 ± 21	1103 ± 22
Total	73,534 ± 1471	86,984 ± 1740
**Carotenoids**		
β-carotene	1 ± 0.1	1 ± 0.1
zeaxanthin	74 ± 7.4	111 ± 11.1
lutein	557 ± 55.7	541 ± 54.1
Total	632 ± 63.2	653 ± 65.3
**Chlorophylls**		
chlorophyll a	3250 ± 325.0	1929 ± 192.9
chlorophyll b	1390 ± 139.0	1015 ± 101.5
Total	4640 ± 464.0	2944 ± 294.4

Phytochemical classes are put in bold.

**Table 4:** Phytochemical content in the extracts of raw vs. fermented *S. scabrum* grown in Normal-greenhouse (Ng) vs. Open-greenhouse (Og) in µg/g DM (location B). The data are presented as phytochemical content ± average analytical error.

Polyphenols	Ng Ethanol	Og Ethanol
tentative structure		
3-caffeoylquinic acid	3815 ± 76	3636 ± 73
5-caffeoylquinic acid	6669 ± 133	15,526 ± 311
4-caffeoylquinic acid	28,790 ± 576	114,700 ± 2294
caffeoylmalate	773 ± 15	n.d.
caffeoylmalate	31,704 ± 634	42,237 ± 845
quercetin-3-glucosylrhamnogalcatoside	2035 ± 41	3096 ± 62
coumaric acid	1501 ± 30	1603 ± 32
quercetin-3-rhamnogalactoside	377 ± 19	627 ± 31
quercetin-3-rhamnosylrhamnogalactoside (isomer 2)	3705 ± 74	4464 ± 89
quercetin-3-rhamnosylrhamnogalactoside (isomer 3)	1620 ± 32	1555 ± 31
sinapoylmalate	522 ± 26	550 ± 28
kaempferol-3-diglucoside	457 ± 9	541 ± 11
kaempferol-3-rhamnosylrhamnogalactoside (isomer 1)	611 ± 31	550 ± 28
quercetin-3-pentosylrutinoside	1037 ± 52	788 ± 39
sinapic acid	1113 ± 22	1106 ± 22
kaempferol-3-rhamnosylrhamnogalactoside (isomer 1)	968 ± 19	1337 ± 27
quercetin-3-rutinoside	2393 ± 48	4128 ± 83
Total	88,090 ± 1762	196,444 ± 3929
**Carotenoids**		
β-carotene	1 ± 0.1	1 ± 0.1
zeaxanthin	24 ± 2.4	65 ± 6.5
lutein	187 ± 18.7	104 ± 10.4
Total	212 ± 21.2	170 ± 17.0
**Chlorophylls**		
chlorophyll a	2870 ± 287.0	1884 ± 188.4
chlorophyll b	856 ± 85.6	539 ± 53.9
Total	3726 ± 372.6	2424 ± 242.4

(n.d. = not detected). Phytochemical classes are put in bold.

**Table 5:** Phytochemical content in the extracts of *S. scabrum* in µg/g DM (location D). The data are presented as phytochemical content ± average analytical error.

Polyphenols	Raw_K Ethanol
tentative structure	
3-caffeoylquinic acid	1899 ± 38
5-caffeoylquinic acid	1064 ± 21
4-caffeoylquinic acid	7826 ± 157
caffeoylmalate	6163 ± 123
caffeoylmalate	33,424 ± 668
quercetin-3-glucosylrhamnogalcatoside	3007 ± 60
coumaric acid	2118 ± 42
quercetin-3-rhamnogalactoside	639 ± 32
quercetin-3-rhamnosylrhamnogalactoside (isomer 2)	5749 ± 115
quercetin-3-rhamnosylrhamnogalactoside (isomer 3)	2722 ± 54
sinapoylmalate	n.d.
kaempferol-3-diglucoside	514 ± 26
kaempferol-3-rhamnosylrhamnogalactoside (isomer 1)	821 ± 41
quercetin-3-pentosylrutinoside	1177 ± 24
sinapic acid	1049 ± 21
kaempferol-3-rhamnosylrhamnogalactoside (isomer 1)	1972 ± 39
quercetin-3-rutinoside	8318 ± 166
Total	78,462 ± 1569
**Carotenoids**	
β-carotene	1 ± 0.1
zeaxanthin	51 ± 5.1
lutein	141 ± 14.1
Total	193 ± 19.3
**Chlorophylls**	
chlorophyll a	2152 ± 215.2
chlorophyll b	672 ± 67.2
Total	2824 ± 282.4

(n.d. = not detected). K means grown in Kenya. Phytochemical classes are put in bold.
